# An interrater reliability analysis of preoperative mortality risk calculators used for elective high-risk noncardiac surgical patients shows poor to moderate reliability

**DOI:** 10.1186/s12871-024-02771-8

**Published:** 2024-10-30

**Authors:** Jacqueline E. M. Vernooij, Lian Roovers, René van der Zwan, Benedikt Preckel, Cor J. Kalkman, Nick J. Koning

**Affiliations:** 1 Department of Anesthesiology, Rijnstate Hospital, Arnhem, The Netherlands; 2 Clinical Research Center, Rijnstate Hospital, Arnhem, The Netherlands; 3 Department of Vital Functions, University Medical Centre Utrecht, Utrecht, The Netherlands; 4 Department of Anesthesiology, Amsterdam University Medical Centre, University of Amsterdam UvA, Meibergdreef 9, Amsterdam, 1105 AZ The Netherlands

**Keywords:** High-risk, Mortality, Perioperative, Calculator, Risk-assessment, Elective surgery

## Abstract

**Background:**

Multiple preoperative calculators are available online to predict preoperative mortality risk for noncardiac surgical patients. However, it is currently unknown how these risk calculators perform across different raters. The current study investigated the interrater reliability of three preoperative mortality risk calculators in an elective high-risk noncardiac surgical patient population to evaluate if these calculators can be safely used for identification of high-risk noncardiac surgical patients for a preoperative multidisciplinary team discussion.

**Methods:**

Five anesthesiologists assessed the preoperative mortality risk of 34 high-risk patients using the preoperative score to calculate postoperative mortality risks (POSPOM), the American College of Surgeons surgical risk calculator (SRC), and the surgical outcome risk tool (SORT). In total, 170 calculations per calculator were gathered.

**Results:**

Interrater reliability was poor for SORT (ICC (C.I. 95%) = 0.46 (0.30–0.63)) and moderate for SRC (ICC = 0.65 (0.51–0.78)) and POSPOM (ICC = 0.63 (0.49–0.77). The absolute range of calculated mortality risk was 0.2–72% for POSPOM, 0–36% for SRC, and 0.4–17% for SORT. The coefficient of variation increased in higher risk classes for POSPOM and SORT. The extended Bland–Altman limits of agreement suggested that all raters contributed to the variation in calculated risks.

**Conclusion:**

The current results indicate that the preoperative risk calculators POSPOM, SRC, and SORT exhibit poor to moderate interrater reliability. These calculators are not sufficiently accurate for clinical identification and preoperative counseling of high-risk surgical patients. Clinicians should be trained in using mortality risk calculators. Also, clinicians should be cautious when using predicted mortality estimates from these calculators to identify high-risk noncardiac surgical patients for elective surgery.

**Supplementary Information:**

The online version contains supplementary material available at 10.1186/s12871-024-02771-8.

## Highlights

-Interrater reliability for POSPOM, SRC and SORT preoperative mortality risk predictors is lower than expected.

-Clinicians need to use preoperative mortality risk calculators with caution when using them for identification and preoperative counseling of high-risk noncardiac surgical patients.

-In this study all raters using POSPOM, SRC and SORT for preoperative mortality risk calculation, contributed to variability in risk estimates.

**Supplementary Information:**

The online version contains supplementary material available at 10.1186/s12871-024-02771-8.

## Introduction

Identifying high-risk patients for perioperative treatment and decision-making remains a challenge due to difficulty in reliably estimating morbidity and mortality risks [1–3]. Assessing surgical risk helps allocating resources, obtaining informed consent, and making shared decisions with a preoperative multidisciplinary team (MDT) [4–6]. Multimorbidity is increasing globally in an ageing population with a growing burden of chronic diseases [7]. It has been shown that high-risk noncardiac surgical patients suffer disproportionally from perioperative complications [1]. Preoperative mortality risk calculators are available and may help to identify elective high-risk noncardiac surgical patients already before surgery, triggering efforts to lower the burden of possible complications, e.g., by extended monitoring or specified treatments. A systematic review suggested a significant risk of bias in developping current preoperative risk calculators due to lack of external validation, highlighting the need for enhanced performance and reliability to ensure their effectiveness in clinical practice [8]. Low reliability and performance, despite their general availability, may be the reason why preoperative risk calculators are not yet consistently used in clinical practice [4, 9–12]. For daily clinical use, good predictive performance, low interrater variability and user friendliness are essential [8, 9, 11, 13]. In addition, discrepancies in predictor measurements can cause miscalibration, changes in discriminatory ability, and overall accuracy, leading to clinically relevant variability in risk calculator results [14–17]. Previous studies have shown that physicians must trust a mortality risk calculator before utilization [18, 19]. High-risk patients suffer especially from complications, and it has been shown that the complications often result in perioperative death [1]. Therefore, adequate preoperative calculation of mortality risk and early recognition of high-risk noncardiac surgical patients could benefit from reliable preoperative risk calculation. Using dependable preoperative mortality risk calculators to identify high-risk patients scheduled for elective surgeries can help establish a comprehensive system for managing high-risk surgical patients within the hospital. Once high-risk patients have been identified and selected, a collaborative approach involving anesthesiologists, surgeons, and other healthcare professionals can be employed to optimize their care. This multidisciplinary team can assess the potential risks and benefits of the planned surgery and consider the patient's preferences to enhance care, minimize complications, and prevent perioperative deaths. Ultimately, the risks will be communicated to the patient following the multidisciplinary decision-making process to facilitate shared decision-making and improve overall care [20].

The current study assessed the consistency among five anesthesiologists in calculating preoperative mortality risk scores for elective high-risk noncardiac surgical patients in clinical practice. Most patients were scheduled for intermediate and low-risk surgeries, but with uncertainty about the potential risks and benefits of the planned surgical procedure. After identification the patients were discussed in a preoperative MDT meeting. All surgeries intended for the high-risk patients under review were standard procedures at the hospital*.* The hypothesis was that the available risk calculators would show moderate to good reliability. For this purpose, the anesthesiologists used the following three risk calculators: the preoperative score to predict postoperative morbidity [POSPOM [21]], the American College of Surgeons surgical risk calculator [SRC [22]], and the surgical outcome risk tool [SORT [23]].

## Methods

### Ethics and registration

The current retrospective reliability study (clinicaltrials.gov: NCT06410183) analyzed the medical records of 34 high-risk noncardiac surgical patients who were discussed in a preoperative multidisciplinary team meeting. The Research Ethics Committee Arnhem/Nijmegen decided that the study did not fall within the remit of the Medical Research Involving Human Subjects Act (WMO). (file number 2019–5154; February 8th, 2019, Prof. Dr. P.N.R. Dekhuijzen). No formal judgement about the rating of anesthesiologists was asked.

The local feasability committee of the Rijnstate Hospital waived the need for informed consent as the data were analyzed retrospectively by members of the treatment team who already had access to the patient data.

Methods and reporting followed the Guidelines for Reporting Reliability and Agreement Studies (GRRAS) [24].

### Inclusion and exclusion criteria

The study included all adult elective high-risk noncardiac surgical patients discussed during a preoperative MDT meeting in 2015 in a teaching hospital where all surgical specialties are executed, excluding neurosurgery, cardiac surgery, and transplant surgery. Missing data in the patient file necessary for perioperative mortality risk prediction was an exclusion criterium.

### Preoperative mortality risk calculators

For the current study, three preoperative mortality risk calculators were chosen that only use preoperative variables to predict postoperative mortality as the primary outcome. We chose these models because preoperative data might provide the most significant decision-related benefit [25]. Moreover, these calculators are freely available online or in app form: POSPOM [21], SRC [22]} and SORT [23] and can already be used in clinical practice.

POSPOM and SORT already have been externally validated on complete cohorts in the past [26–29]. For SRC, external validations have only been performed for single specialties and not for complete noncardiac surgery cohorts.

POSPOM calculates in-hospital mortality risk, while SRC and SORT estimate 30-day postoperative mortality risk. The correlation coefficients between 30-day and in-hospital mortality are reasonable and calculated results can be compared [30].

The calculators of POSPOM, SRC and SORT can be found on their websites (reference http://perioperativerisk.com/mortality, https://riskcalculator.facs.org/RiskCalculator and [31]).

### Setting

An anesthesiologist or anesthesiology resident screened the preoperative patient and recommended further exams or consultations based on guidelines [32, 33]. According to Dutch preoperative guidelines, patients were selected for an MDT discussion at the preoperative screening clinic if the combination of comorbidities and surgical procedure led to doubt on the harm-benefit ratio for the patient undergoing surgery [20, 34]. During an MDT meeting, an anesthesiologist, a surgeon, and at least one other relevant medical specialist discussed the intended surgerys’ harm-benefit ratio for the patient concerning the patients’ wishes, optimal preoperative preparation, and potential alternatives based on the patient's health status [20].

### Raters

Five consultant anesthesiologists, with 1 to 30 years of clinical experience, were invited to participate in this research. Prior experience with preoperative mortality risk calculation was not required, and none of the anesthesiologists used it on a regular basis. The five consultant anesthesiologists calculated the mortality risks associated with noncardiac surgeries in the respective high-risk adult patients independently. Each consultant was guided through navigating the calculators’ functionality without formal training to ensure a realistic, contemporary, clinical scenario. We did not instruct the raters on missing data, we left it to the raters’ decision how to fill in the calculator in order to reflect clinical practice. The consultants were provided with all relevant information collected during the preoperative visit and from consultations with medical specialists. Importantly, they used only this information, without any follow-up or additional patient data. The information included demographic data, patient comorbidities, medication, ASA physical status score, further details from consultant specialists, and planned surgical treatment. The anesthesiologists were instructed to use only the available information while calculating the respective risk.

### Data collection

The anesthesiologist raters collected the information needed for the calculations from a copy of the patient's health forms used in clinical practice. The results of the calculations from the three risk calculators and the filled-in forms (with all the necessary variables) were copied from the Internet and sent to the principal investigator (JV) by email.

### Outcome

The primary outcome of this study was the interrater reliability of three preoperative mortality risk calculators: POSPOM, SRC and SORT. Secondary outcomes were the agreement between raters per variable, patient characteristics, and 30-day (or in-hospital) perioperative or peri-MDT discussion mortality for patients who did not undergo surgery.

### Sample size

In this study, power calculation was not performed due to the exploratory nature of the research. The sample size was based on a one-year cohort of MDT discussed high-risk noncardiac surgical patients. The number of raters in this study was not determined through a power calculation either. Instead, the number of raters was based on practical considerations.

### Statistical analysis

Statistical analysis was performed using R statistical software version 4.1.1. R Core Team (2021). Continuous variables are summarized as either the means ± standard deviations or the medians and interquartile ranges, as appropriate. We calculated interrater variability using the intraclass correlation (ICC) for the three calculators. Since all predictors were binary or categorical, interrater reliability for each predictor was computed using Fleiss’ kappa [35]. Confidence intervals were calculated via bootstrap percentile. Fleiss' kappa couldn't be determined if predictors showed (near) complete agreement [36]. Fleiss' kappa and ICC values less than 0.5 were considered poor reliability, values between 0.5 and 0.75 as moderate reliability, values between 0.75 and 0.9 as good reliability, and values greater than 0.90 as excellent reliability. Agreement per predictor for every calculator was calculated in percentages and (near) complete agreement per patient. A near complete agreement was defined as > 82% agreement [35].

The patients were grouped in mortality risk groups (< 1%; 1–2,5%; 2,6–5%; 5,1–10% and > 10%) as described by Wong et al. [3] (adapted for the higher classes because of limited data) and mean and median risks were determined per risk group. Subsequently, the mean variance and the variation coeficient per risk group per calculator were determined. In addition, extended Bland–Altman limits of agreement analyses were performed to graphically present agreement between the five raters [37].

## Results

Based on the study protocol eight patient files had to be excluded due to incomplete data or because the patients were younger than 18 years. Thirty-four patients were included. In total, 170 calculations per calculator were gathered.

### Patient characteristics

Patient characteristics are shown in Table 1. Thirty-one patients (91%) had an American Society of Anesthesiology Physical Status (ASA-PS) class of 3 or 4. The only ASA-PS 1 patient was a Jehovah's Witness with a molar pregnancy scheduled for termination. A 92-year-old female patient with an ASA-PS score of 2 was scheduled for a total hip replacement and suffered from persistent anemia. The second patient with an ASA-PS 2 classification was pregnant and suffered from cholecystitis following gastric bypass surgery. Sixteen patients underwent surgery, whereas 18 received nonsurgical care after the MDT discussion. Thirty-day mortality rate was zero for those patients who underwent surgery and 3% (1 patient) for those who did not.

**Table 1:** Characteristics of patients for whom mortality risks were calculated

	*N* = 34
Age years median/(IQR)	69.5 (65–77)
Male N (%)	16 (47)
ASA PS (1/2/3/4)	1/2/11/20
Surgery /no surgery	16/18 (47/53)
Type of surgery;	N (%)
- Major abdominal	4 (12)
- Minor abdominal	1 (3)
- Urologic	8 (2)
- Vascular	3 ((9)
- Thoracic	4 (12)
- Orthopedic	5 (15)
- Other surgery	12(35)
Pre-existing conditions	
- Chronic heart failure	20 (59)
- Coronary artery disease	17 (50)
- Hypertension	16 (47)
- Cancer (last year)	13 (38)
- COPD (Gold class 1/2/3/4) (5%)	10 (2/2/2/4) (29)
- Arrhythmia	8 (24)
- TIA/CVA	6 (18)
- NIDDM	5 (15)
- Chronic renal failure	4 (12)
- Epilepsy	3 (9)
- IDDM	2 (6)
- Dementia	2 (6)

*ASA PS score* American Society of anesthesiologists physical status score, *TIA/CVA* Transient ischemic attack/cerebrovascular accident, *(N)IDDM* (Non) insulin dependent diabetes mellitus, *COPD *Chronic obstructive pulmonary disease

### Interrater reliability of calculators

**Fig. 1:**
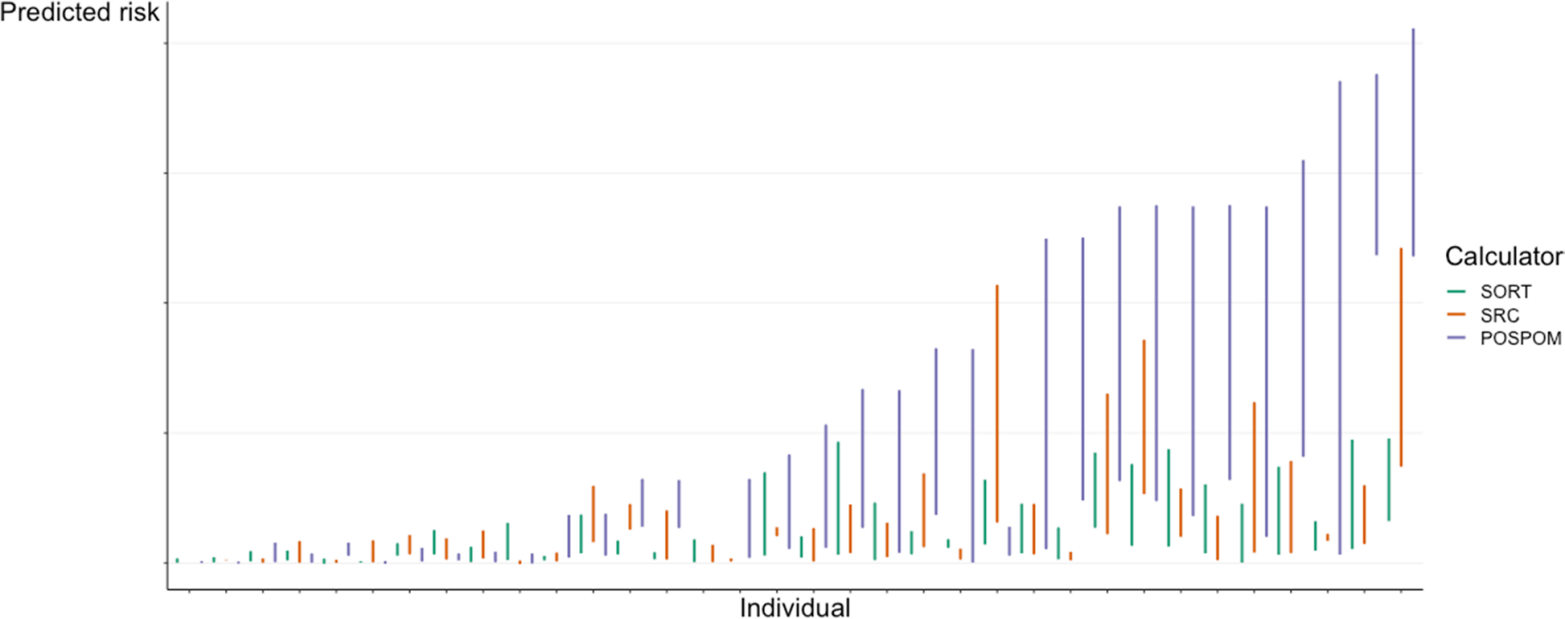
Variability in predicted mortality risks per calculator per individual patient. Figure 1 shows calculations per patient by 5 raters on the X-axis, the length of the line (Y-axis) shows the range of the ratings between the raters. Green calculations: surgical outcome risk tool SORT, orange calculations: surgical risk calculator SRC, and purple calculations: preoperative tool to calculate postoperative mortality POSPOM

Figure 1 (and Additional file/Fig. 1) shows the variation in calculated risks for each individual (patient) per calculator. The intraclass correlation (ICC) (C.I. 95%) was moderate for POSPOM = 0.63 (0.49–0.77) and SRC = 0.65 (0.51–0.78) and poor for SORT: ICC = 0.46 (0.30–0.63).

The study found that there was a near-complete agreement [35] for predictors between the raters in a limited number of patients: for the predictors of POSPOM, there was near-complete agreement in 24% of the predictors, for SRC in 45%, and for SORT in 13% of predictors. (Additional file/Table 2).

### Interrater reliability for variables used in POSPOM, SRC and SORT

The interrater reliability per predictor between the anesthesiologists, measured by the Fleiss' kappa, ranged from poor to good for POSPOM and SORT and from fair to good for SRC. The percentage of predictors that showed good to excellent reliability (κ > 0.75) was highest for SORT: 33% (Additional file/Table 2). The predictors with the highest Fleiss' kappa (95% Confidence Index: (C.I.)) were for SRC: gender (κ = 0.83: 0.72–0.94); for SORT: age (κ = 0.79: 0.64–0.90) and high-risk surgery: (κ = 0.79; 0.65–0.93) and for POSPOM: surgery type (κ = 0.73: 0.63–0.82). The predictors with the lowest Fleiss' kappa were for SORT: the urgency of the procedure ( κ = 0.17: 0.05–0.30); for POSPOM: chronic respiratory failure (κ = 0.17: 0.00–0.36) and for SRC: dyspnea (κ = 0.26: 0.13–0.39).

### Variance

The results indicated that the highest mean–variance was observed in the highest risk classes across all calculators, as demonstrated in Table 2. In contrast, SORT exhibited the lowest mean–variance, whereas POSPOM showed the highest.

**Table 2:** Classes of risk per risk calculator with mean calculated risks, mean coefficient of variation and mean variance per class

Risk calculator	POSPOM				SRC	n			SORT			
Risk classes	Mean Risk	n	CV	Mean Variance	Mean Risk	n	CV	Mean Variance	Mean Risk	n	CV	Mean Variance
< 1%	0.2	5	0.85	0.2	0.4	7	0.79	0.1	0.5	6	0.39	3.3
1.1–2.5	1.6	7	0.53	2.8	1.8	9	0.41	1.7	1.7	11	0.57	2.6
2.6–5	4	5	0.98	124.4	3.5	4	0.15	3.9	3.7	9	0.77	10.9
5.1–10	8.6	8	0.74	107.6	6.6	10	0.16	19	8.6	7	0.69	30
> 10	34.1	9	0.63	369.2	22.7	4	0.28	155.3	15.6	1	*	56.4

*POSPOM* Preoperative score to predict postoperative mortality, *SRC* Surgical risk calculator, *SORT* Surgical outcome risk tool, *CV* Coefficient of variety, * only one calculation, *n* Number of patients per risk class

### Limits of agreement

**Fig. 2:**
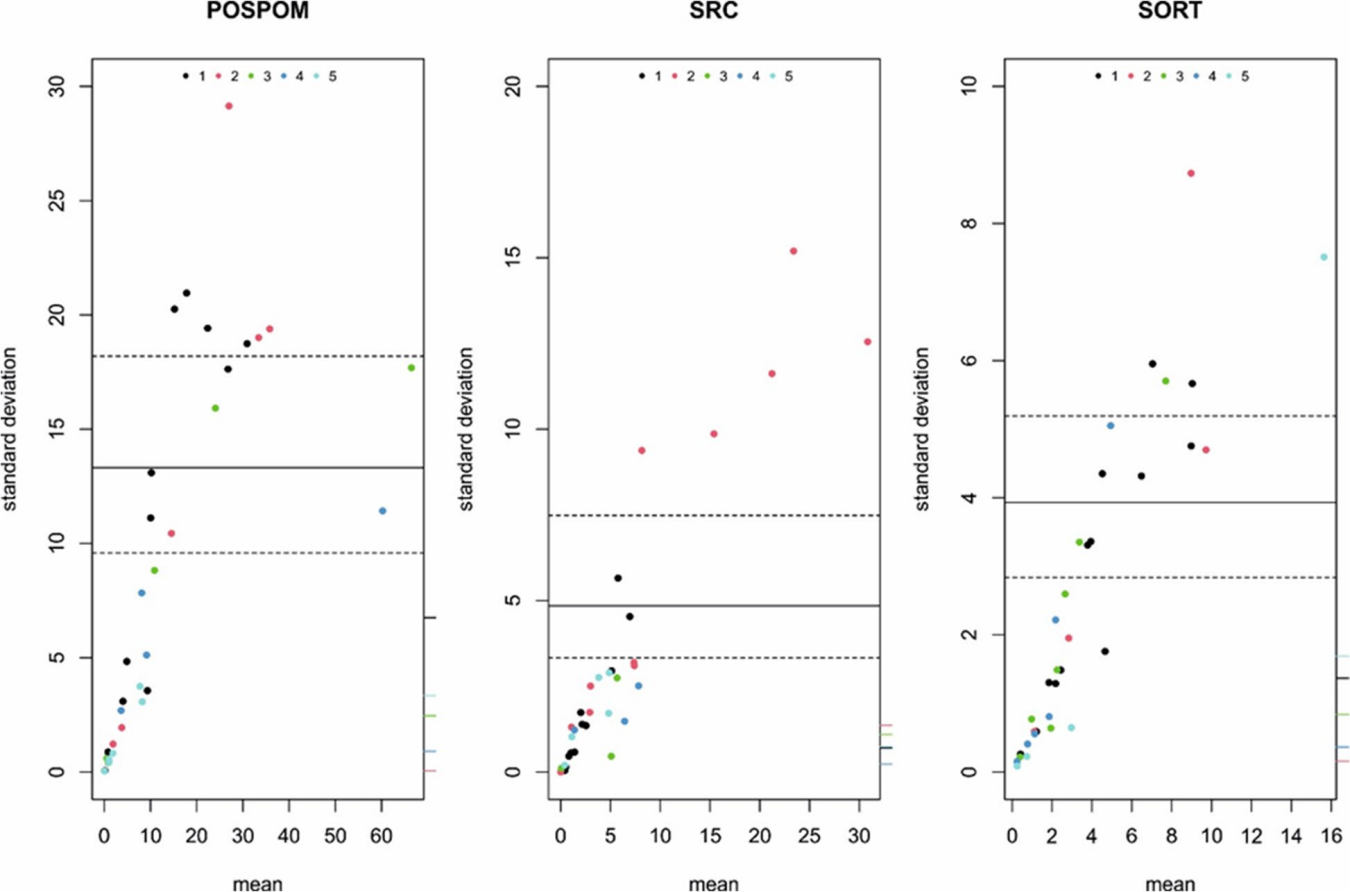
Extended Bland–Altman limits of agreement plots for 5 raters of preoperative mortality risks. Explanation: Dark line: Limits of agreement (LOA); Grey dotted line: 95% confidence intervals for upper and lower limit of agreement for the LOA; Colored dots: every rater has a color, the dots show the rater responsible for the largest deviation from the intrasubject mean; Tick marks on the right y-axis: absolute rater bias

The Bland–Altman limit of agreement plots (Fig. 2) for POSPOM and SORT revealed bias and heterogeneity among patients with higher risk calculations. SRC was found to show less bias and heterogeneity between the raters than POSPOM. The extended Bland–Altman limits of agreement suggested that all raters contributed to the variation in mortality risk.

## Discussion

The current study is, to our knowledge, the first to compare and investigate three preoperative mortality risk calculators. Our results show moderate ICC for POSPOM and SRC, and poor for SORT. The interrater reliability per predictor ranged from poor to good (Fleiss’ kappa scores) for POSPOM and SORT and from fair to good for SRC. Moreover, bias and heterogeneity among raters were detected especially for the patients in the higher high-risk patient classes.

In this study, we focused on high-risk surgical patients. It seems reasonable to centralize high-risk procedures (e.g., oesophagectomy, liver surgery, pancreatic surgery, rectal surgery etc.) in selected centers to ensure sufficient expertise, leading to better outcomes [38, 39]. In small countries centralisation is easier to organize regarding the burden of travel for patients and their families [40]. In large countries though, programs should be established to lessen the burden of travel on patients and their families as much as possible [40–42].

For high-risk patients no centralized care exists. Therefore, it is important to improve recognition of and care for high-risk patients in all hospitals.

Given the considerable variability in risk prediction values and the substantial confidence intervals, these three risk calculators are currently not considered accurate for preoperative patient counseling, specifically for high-risk patients. Reliable, local risk models that capture the performance of local surgeons and healthcare systems may offer better suitability for preoperative risk assessments, although their comprehensive evaluation and validation are still pending.

One finding of this study is that human errors occurred quite frequently during data entry. The extended Bland–Altman Limits of agreement plots showed that all raters made errors. Barchard and Pace investigated the impact of human errors in data entry on research [43]. The current study also revealed that interpretable cardiac and pulmonary function information predictors were sources of variability, for example, for variables such as dyspnea, chronic respiratory failure, and congestive heart disease. By definition, a high-risk patient has numerous long-term health issues. Each variable requiring interpretation contributes to the variability in calculating mortality risk. Other studies identified errors with data entry as well with the mortality risk prediction models p-POSSUM [44] and SRC [22, 45, 46]. Shiloach et al. showed that a specialized training program regarding data entry for SRC significantly improved the proficiency of the audit raters, resulting in a more reliable data collection [46]. In the current era it seems more logical to respond to digital and/or artificial intelligence techniques to tackle data entry errors. Due to this study's retrospective nature, age had to be recalculated and categorized at the time of the study, potentially leading to variability between physicians. Conducting prospective mortality risk calculation at the preoperative visit could reduce inter-rater variability, particularly if age is already available in the electronic health record and does not need to be manually inputted. Calculating gender variability is unnecessary, and any observed variability in gender is likely the result of data entry errors. Therefore, mortality risk calculators should be integrated into electronic health records (EHRs) to reduce the potential for human errors and misclassifications. These calculators should primarily use digital, numerical patient data to minimize the need for human transcription or data categorization. Nevertheless, even with the availability of risk calculators in the EHR, there is a possibility of variability in the physician's interpretation of the predictor's grade or level if interpretable variables are crucial for risk calculation.

Identifying and discussing elective high-risk patients before surgery is crucial for evaluating the benefits and risks of surgery. A preoperative MDT meeting can optimize patient health, minimize complications, and allocate resources efficiently [20]. It is crucial to identify the appropriate patients who may benefit from a preoperative multidisciplinary discussion since organizing and conducting an MDT meeting requires significant time and resources. When using a 30-day 5% mortality risk as the threshold for defining high-risk patients in this study (a 5% 30-day mortality risk is also used to delineate high-risk surgery), it was discovered that more than half of the patients identified as high-risk patients by anesthesiologists in the current study during a preoperative assessment did not meet the criteria for a high-risk status (Table 2). It has been suggested that adding the anesthesiologists’ judgment to the risk calculator (e.g., subjective SORT) may improve the identification of high-risk noncardiac surgical patients [3].

One of the reasons for not using preoperative risk calculators in clinical practice is the lack of trust in the reliability of the existing calculators [47]. Unreliability could, next to interrater variability, also follow from imperfect measurements of predictors, [15] improper development of the calculator, [8] or inexperience in calculating mortality risk. Improving access to risk calculators helps, but mandatory training for clinicians in the use of preoperative mortality risk calculators is necessary to ensure proficiency for clinicians even if the calculators are built into the EHR.

It is conceivable that inaccurate predictions are not caused by clinical process measures, but rather by the subjective interpretation of variable grades. Nonetheless, it is known that process measures in the development, calibration and external validation of risk prediction models contribute to inaccurate predictions. Existing prediction models' external validation, calibration and updating processes are resource-intensive and not conducted frequently. Nevertheless, experts in prediction modeling recommend enhancing existing models instead of creating new ones from scratch [48–50].

This study highlights the need for improved risk calculators that use fewer or none of the interpretable predictors and are less prone to interrater variability. Sound clinical usability is another prerequisite for the increased use of preoperative risk calculators [8]. Improved risk assessments and documentation will not directly enhance outcomes. However, better risk assessments can improve the identification of elective high-risk noncardiac surgical patients and lead to a greater number of patients receiving multidisciplinary, personalized care in the Netherlands. This multidisciplinary, customized care will enhance the treatment of the individual high-risk patient by reducing adverse outcomes, optimizing care, and improving shared decision-making with these patients.

The study found a previously unreported interrater variability with the three preoperative mortality risk predictors. Future research should focus on reducing subjectivity in predictors used in preoperative mortality risk calculators. Also, as highlighted by Mathiszig-Lee et al., prioritizing the quantification of and incorporation of uncertainty into calculated risks may enrich multidisciplinary team discussions, enhance risk communication, and improve the process of obtaining informed consent from the patient [51].

### Limitations

Although the current size of the patient cohort limits the study, the current high-risk population includes patients whose risk calculations are most crucial for preoperative shared decision-making. Additionally, the survey was conducted retrospectively, and the anesthesiologist did not evaluate the patients in person. However, post-pandemic, modern practice involves electronic screening, and the anesthesiologist who provides anesthesia is usually not involved in the screening process [52]. Thirdly, the lack of training may have caused variability among raters but reflects the current use of preoperative risk calculators.

Our research findings indicate a high level of agreement among the raters for various variables from POSPOM and SRC. However, the agreement was so near-complete that it was impossible to determine Fleiss kappa [36]. This high level of agreement among raters was mainly for variables that were not frequently observed in the patient cohort, including dementia, hemiplegia, ventilator dependency, use of steroids, chronic renal failure, and chronic hemodialysis. Logically, these variables did not appear to contribute significantly to the observed inter-rater variability.

## Conclusion

The current study suggests that anesthesiologists need to have more consistent agreement when using POSPOM, SRC, and SORT to assess mortality risk in elective noncardiac surgical patients. Accurate preoperative risk assessments are crucial for identifying high-risk noncardiac surgical patients undergoing elective procedures. The current preoperative mortality risk calculators are not sufficiently reliable in identifying these high-risk patients. It is essential to improve these calculators' reliability, accuracy, and usability to improve preoperative counseling and multidisciplinary decision-making for these patients before surgery. It is imperative to train clinicians in the correct use of preoperative mortality risk calculators.

## Supplementary Information

Supplementary Material 1.

Supplementary Material 2.

## Data Availability

Data are available on request. Send an email to Jacqueline Vernooij at jvernooij@rijnstate.nl

## Abbreviations

POSPOM: Preoperative score to calculate postoperative mortality
SRC: American College of Surgeons surgical risk calculator
SORT: Surgical outcome risk tool
MDT: Multidisciplinary team
ICC: Intraclass correlation coefficient
ASA ps score: American Society of Anaesthesiologists physical status score
