# Supplementary material for: An interrater reliability analysis of preoperative mortality risk calculators used for elective high-risk noncardiac surgical patients shows poor to moderate reliability
Source: BMC Anesthesiol. 2024 Oct 30;24:392. doi: 10.1186/s12871-024-02771-8 (PMC11523836; doi:10.1186/s12871-024-02771-8)
Supplement: Supplementary file 1 — Supplementary Material 1. [file 12871_2024_2771_MOESM1_ESM.docx]

**Additonal file 1: POSPOM** mortality risk prediction (fraction) for 34 patients calculated by 5 anesthesiologists. Note: the scales of the calculated risks vary per calculator.


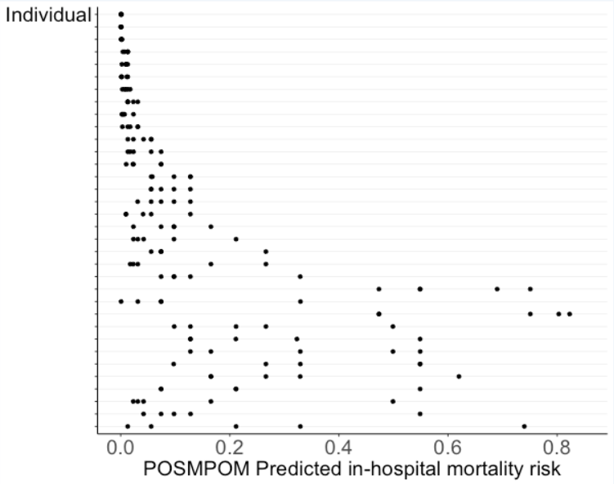


The x-axis shows the mortality calculations of five raters, while the y-axis displays the 34 individual high-risk patients.

**SRC** mortality risk prediction (fraction) for 34 patients calculated by 5 anesthesiologists

**
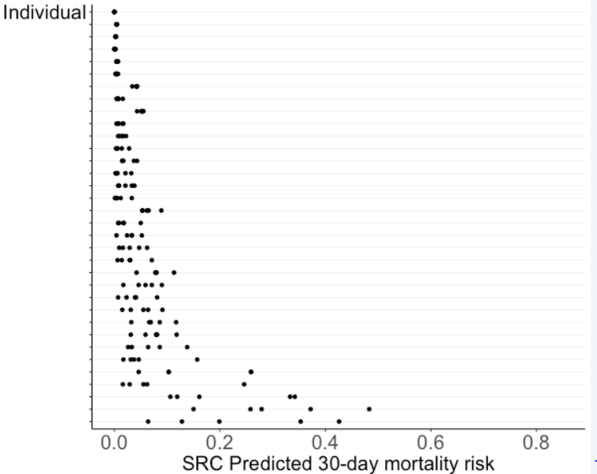
**

The x-axis shows the mortality risk fractions of five raters, while the y-axis displays the 34 individual, high-risk patients.

**SORT** mortality risk prediction (fraction) for 34 patients calculated by 5 anesthesiologists

**
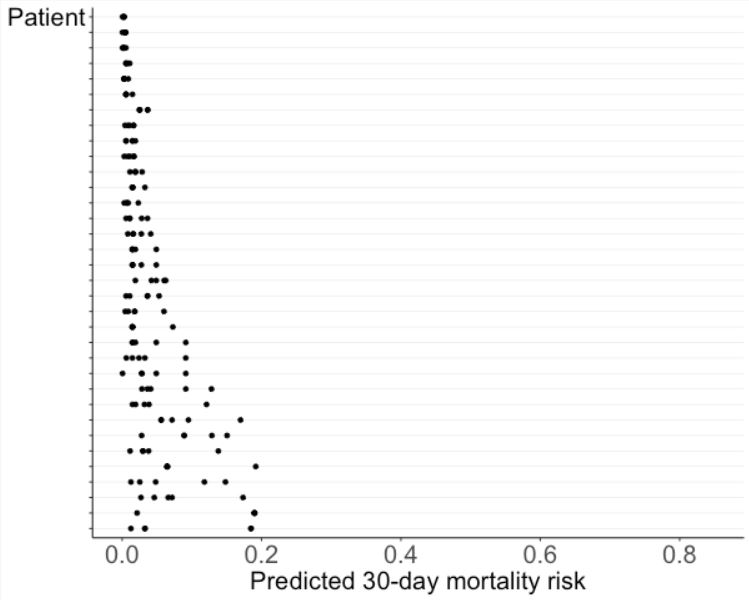
**

The x-axis shows the mortality risk fractions of five raters, while the y-axis displays the 34 individual, high-risk patients.
