# Supplementary material for: An interrater reliability analysis of preoperative mortality risk calculators used for elective high-risk noncardiac surgical patients shows poor to moderate reliability
Source: BMC Anesthesiol. 2024 Oct 30;24:392. doi: 10.1186/s12871-024-02771-8 (PMC11523836; doi:10.1186/s12871-024-02771-8)
Supplement: Supplementary file 2 — Supplementary Material 2. [file 12871_2024_2771_MOESM2_ESM.docx]

**Additional file 2: Fleiss’ kappa per predictor and number of near complete (≥82%) agreement in scoring between 5 anesthesiologists for POSPOM, SRC and SORT**

| **POSPOM** | **Fleiss’ kappa (C.I. 95%)** | **No of patients with near complete agreement (****≥82%)**  **(5 anesthesiologists) per predictor (%)** |
| --- | --- | --- |
| Surgery type | 0.73 (0.63-0.82) | 17 (50) |
| Age (per 5 years) | 0.52 (0.43-0.61) | 7 (21) |
| Ischemic heart disease | 0.50 (0.34-0.67) | 17 (50) |
| Arrythmia | 0.45 (0.24-0.64) | 18 (53) |
| CHF or cardiomyopathy | 0.69 (0.55-0.83) | 23 (68) |
| Peripheral vascular disease | 0.43 (0.16-0.60) | 23 (68) |
| Cerebrovascular disease | 0.52 (0.25-0.73) | 23 (68) |
| COPD | 0.68 (0.49-0.85) | 25 (74) |
| Dementia | -* | 30 (88) |
| Hemiplegia | -* | 33 (97) |
| Chronic respiratory failure | 0.17 (0.00-0.36) | 12 (35) |
| Chronic alcohol abuse | 0.25 (0.03-0.44) | 23 (68) |
| Active Cancer | 0.35 (0.15-0.58) | 20 (59) |
| Diabetes | 0.47 (0.30-0.63) | 15 (44) |
| Transplanted organ | -* | 32 (94) |
| Chronic renal failure | 0.38 (0.15-0.58) | 17 (50) |
| Chronic Hemodialysis | -* | 33 (97) |
|  |  |  |
| Percentage of good to excellent Fleiss’ kappa (>0.75) | 0% | 24% |

POSPOM: preoperative score to predict postoperative mortality; CHF: Chronic Heart Failure; COPD: Chronic Obstructive Pulmonary Disease; *: almost complete or complete agreement: no Fleiss’ kappa can be calculated

| **SRC** | **Fleiss’ kappa (C.I. 95%)** | **No of patients with near complete agreement (≥82%)**  **(5 anesthesiologists) per predictor** |
| --- | --- | --- |
| CPT code | 0.34 (0.21-0.42) | 5 (15) |
| Age (4 categories) | 0.62 (0.47-0.62) | 18 (53) |
| Sex | 0.84 (0.72-0.94) | 27 (79) |
| Functional status | 0.37 (0.21-0.54) | 8 (24) |
| Emergency | -* | 30 (88) |
| ASA PS | 0.50 (0.33-0.67) | 17 (50) |
| Steroid use | -* | 32 (94) |
| Ascites 40 days | -* | 32 (94) |
| Sepsis 48 days | -* | 34 (100) |
| Ventilator dependency | -* | 34 (100) |
| Disseminated cancer | -* | 29 (85) |
| Diabetes | 0.60 (0.42-0.77) | 17 (50) |
| Hypertension with medication | 0.53 (0.35-0.69) | 19 (56) |
| Congestive Heart failure | 0.56 (0.38-0.71) | 19 (56) |
| Dyspnea | 0.26 (0.13-0.39) | 5 (15) |
| Smoker 1 year | 0.69 (0.53-0.86) | 24 (71) |
| Severe COPD | 0.49 (0.30-0.69) | 23 (68) |
| Dialysis | -* | 33 (97) |
| AKI | -* | 34 (100) |
| BMI | 0.82 (0.71-0.91) | 24 (71) |
|  |  |  |
| Percentage of good to excellent Fleiss’ kappa  (>0.75) | 10 % | 45% |

SRC: surgical risk calculator; CPT code: Current Procedural Terminology code; ASA PS: American Society of Anesthesiologists physical status; COPD: Chronic Obstructive Pulmonary Disease; AKI: Acute Kidney Injury; BMI: Body Mass Index; *: almost complete or complete agreement: no Fleiss’ kappa can be calculated

| **SORT** | **Fleiss’ kappa (C.I. 95%)** | **No of patients with near complete agreement (≥82%)**  **(by 5 anesthesiologists) per predictor** |
| --- | --- | --- |
| ASA PS | 0.45 (0.30-0.60) | 11 (32) |
| Procedure severity | 0.51 (0.39-0.64) | 11 (32) |
| Urgency | 0.18 (0.05-0.30) | 7 (21) |
| Thoracic/vascular/  Gastrointestinal procedure | 0.79 (0.65-0.93) | 28 (82) |
| Active Cancer during last 5 year | 0.50 (0.32-0.68) | 22 (65) |
| Age (<65, 65-80, >80 year) | 0.79 (0.64-0.90) | 26 (76) |
|  |  |  |
| Percentage of good to excellent Fleiss’ kappa  (> 0.75) | 33% | 17% |

SORT: surgical risk calculator; ASA PS: American Society of Anesthesiologists physical status; *: almost complete or complete agreement: no Fleiss’ kappa can be calculated
